# Supplementary material for: HIVprotI: an integrated web based platform for prediction and design of HIV proteins inhibitors
Source: J Cheminform. 2018 Mar 9;10:12. doi: 10.1186/s13321-018-0266-y (PMC5845081; doi:10.1186/s13321-018-0266-y)
Supplement: Supplementary file 2 — Additional file 2. Source code of HIVProtI web server. [file 13321_2018_266_MOESM2_ESM.zip › HIVprotI_Source-code/hivprotI/weka-3-6-6/doc/weka/filters/unsupervised/attribute/Remove.html]

Remove


---


|  |  |  |
| --- | --- | --- |
| **Overview** \| **Package** \| **Class** \| **Tree** \| **Deprecated** \| **Index** \| **Help** \| **Weka's home** | |  |
| **PREV CLASS**   **NEXT CLASS** | **FRAMES**    **NO FRAMES**     **All Classes** |  |
| SUMMARY: NESTED \| FIELD \| CONSTR \| METHOD | DETAIL: FIELD \| CONSTR \| METHOD |  |


---


## weka.filters.unsupervised.attribute Class Remove

```
java.lang.Object
  weka.filters.Filter
      weka.filters.unsupervised.attribute.Remove
```

**All Implemented Interfaces:**: java.io.Serializable, CapabilitiesHandler, OptionHandler, RevisionHandler, StreamableFilter, UnsupervisedFilter

---

``` public class Remove extends Filter implements UnsupervisedFilter, StreamableFilter, OptionHandler ```

A filter that removes a range of attributes from the dataset. Will
re-order the remaining attributes if invert matching sense is turned
on and the attribute column indices are not specified in ascending
order.


Valid options are:

```
 -R <index1,index2-index4,...>
  Specify list of columns to delete. First and last are valid
  indexes. (default none)
```

```
 -V
  Invert matching sense (i.e. only keep specified columns)
```

**Version:**
:   $Revision: 7107 $

**Author:**
:   Len Trigg (trigg@cs.waikato.ac.nz)

**See Also:**: Serialized Form

---

**Constructor Summary**

`Remove()`
  
          Constructor so that we can initialize the Range variable properly.


| **Method Summary** | |
| --- | --- |
| `java.lang.String` | `attributeIndicesTipText()`             Returns the tip text for this property |
| `java.lang.String` | `getAttributeIndices()`             Get the current range selection. |
| `Capabilities` | `getCapabilities()`             Returns the Capabilities of this filter. |
| `boolean` | `getInvertSelection()`             Get whether the supplied columns are to be removed or kept |
| `java.lang.String[]` | `getOptions()`             Gets the current settings of the filter. |
| `java.lang.String` | `getRevision()`             Returns the revision string. |
| `java.lang.String` | `globalInfo()`             Returns a string describing this filter |
| `boolean` | `input(Instance instance)`             Input an instance for filtering. |
| `java.lang.String` | `invertSelectionTipText()`             Returns the tip text for this property |
| `java.util.Enumeration` | `listOptions()`             Returns an enumeration describing the available options. |
| `static void` | `main(java.lang.String[] argv)`             Main method for testing this class. |
| `void` | `setAttributeIndices(java.lang.String rangeList)`             Set which attributes are to be deleted (or kept if invert is true) |
| `void` | `setAttributeIndicesArray(int[] attributes)`             Set which attributes are to be deleted (or kept if invert is true) |
| `boolean` | `setInputFormat(Instances instanceInfo)`             Sets the format of the input instances. |
| `void` | `setInvertSelection(boolean invert)`             Set whether selected columns should be removed or kept. |
| `void` | `setOptions(java.lang.String[] options)`             Parses a given list of options. |

**Methods inherited from class weka.filters.Filter**

`batchFilterFile, batchFinished, filterFile, getCapabilities, getOutputFormat, isFirstBatchDone, isNewBatch, isOutputFormatDefined, makeCopies, makeCopy, numPendingOutput, output, outputPeek, toString, useFilter, wekaStaticWrapper`

**Methods inherited from class java.lang.Object**

`equals, getClass, hashCode, notify, notifyAll, wait, wait, wait`

**Constructor Detail**

### Remove

```
public Remove()
```

:   Constructor so that we can initialize the Range variable properly.


**Method Detail**

### listOptions

```
public java.util.Enumeration listOptions()
```

:   Returns an enumeration describing the available options.

    :   **Specified by:**: `listOptions` in interface `OptionHandler`
    :   **Returns:**: an enumeration of all the available options.

---


### setOptions

```
public void setOptions(java.lang.String[] options)
                throws java.lang.Exception
```

:   Parses a given list of options.


    Valid options are:

    ```
     -R <index1,index2-index4,...>
      Specify list of columns to delete. First and last are valid
      indexes. (default none)
    ```

    ```
     -V
      Invert matching sense (i.e. only keep specified columns)
    ```

    :   **Specified by:**: `setOptions` in interface `OptionHandler`
    :   **Parameters:**: `options` - the list of options as an array of strings **Throws:**: `java.lang.Exception` - if an option is not supported

---


### getOptions

```
public java.lang.String[] getOptions()
```

:   Gets the current settings of the filter.

    :   **Specified by:**: `getOptions` in interface `OptionHandler`
    :   **Returns:**: an array of strings suitable for passing to setOptions

---


### getCapabilities

```
public Capabilities getCapabilities()
```

:   Returns the Capabilities of this filter.

    :   **Specified by:**: `getCapabilities` in interface `CapabilitiesHandler` **Overrides:**: `getCapabilities` in class `Filter`
    :   **Returns:**: the capabilities of this object **See Also:**: `Capabilities`

---


### setInputFormat

```
public boolean setInputFormat(Instances instanceInfo)
                       throws java.lang.Exception
```

:   Sets the format of the input instances.

    :   **Overrides:**: `setInputFormat` in class `Filter`
    :   **Parameters:**: `instanceInfo` - an Instances object containing the input instance structure (any instances contained in the object are ignored - only the structure is required). **Returns:**: true if the outputFormat may be collected immediately **Throws:**: `java.lang.Exception` - if the format couldn't be set successfully

---


### input

```
public boolean input(Instance instance)
```

:   Input an instance for filtering. Ordinarily the instance is processed
    and made available for output immediately. Some filters require all
    instances be read before producing output.

    :   **Overrides:**: `input` in class `Filter`
    :   **Parameters:**: `instance` - the input instance **Returns:**: true if the filtered instance may now be collected with output(). **Throws:**: `java.lang.IllegalStateException` - if no input structure has been defined.

---


### globalInfo

```
public java.lang.String globalInfo()
```

:   Returns a string describing this filter

    :   **Returns:**: a description of the filter suitable for displaying in the explorer/experimenter gui

---


### invertSelectionTipText

```
public java.lang.String invertSelectionTipText()
```

:   Returns the tip text for this property

    :   **Returns:**: tip text for this property suitable for displaying in the explorer/experimenter gui

---


### getInvertSelection

```
public boolean getInvertSelection()
```

:   Get whether the supplied columns are to be removed or kept

    :   **Returns:**: true if the supplied columns will be kept

---


### setInvertSelection

```
public void setInvertSelection(boolean invert)
```

:   Set whether selected columns should be removed or kept. If true the
    selected columns are kept and unselected columns are deleted. If false
    selected columns are deleted and unselected columns are kept.

    :   **Parameters:**: `invert` - the new invert setting

---


### attributeIndicesTipText

```
public java.lang.String attributeIndicesTipText()
```

:   Returns the tip text for this property

    :   **Returns:**: tip text for this property suitable for displaying in the explorer/experimenter gui

---


### getAttributeIndices

```
public java.lang.String getAttributeIndices()
```

:   Get the current range selection.

    :   **Returns:**: a string containing a comma separated list of ranges

---


### setAttributeIndices

```
public void setAttributeIndices(java.lang.String rangeList)
```

:   Set which attributes are to be deleted (or kept if invert is true)

    :   **Parameters:**: `rangeList` - a string representing the list of attributes. Since the string will typically come from a user, attributes are indexed from 1. eg: first-3,5,6-last

---


### setAttributeIndicesArray

```
public void setAttributeIndicesArray(int[] attributes)
```

:   Set which attributes are to be deleted (or kept if invert is true)

    :   **Parameters:**: `attributes` - an array containing indexes of attributes to select. Since the array will typically come from a program, attributes are indexed from 0.

---


### getRevision

```
public java.lang.String getRevision()
```

:   Returns the revision string.

    :   **Specified by:**: `getRevision` in interface `RevisionHandler` **Overrides:**: `getRevision` in class `Filter`
    :   **Returns:**: the revision

---


### main

```
public static void main(java.lang.String[] argv)
```

:   Main method for testing this class.

    :   **Parameters:**: `argv` - should contain arguments to the filter: use -h for help


---


|  |  |  |
| --- | --- | --- |
| **Overview** \| **Package** \| **Class** \| **Tree** \| **Deprecated** \| **Index** \| **Help** \| **Weka's home** | |  |
| **PREV CLASS**   **NEXT CLASS** | **FRAMES**    **NO FRAMES**     **All Classes** |  |
| SUMMARY: NESTED \| FIELD \| CONSTR \| METHOD | DETAIL: FIELD \| CONSTR \| METHOD |  |


---
